# Addition of anterolateral ligament reconstruction to primary hamstring autograft ACLR improves objective rotatory stability and reduces re-tear rates: a systematic review and meta-analysis

**DOI:** 10.1186/s43019-026-00327-3

**Published:** 2026-06-16

**Authors:** Assala Abu Mukh, Elsayed Ahmed Abdelatif, Shengdong Yang, Hyeon Seok Hong, Lael Kang, Hye Chang Rhim, Ki-Mo Jang

**Affiliations:** 1 Korea University College of Medicine, Korea University, Seoul, 02841 Korea, Republic of; 2 Department of Orthopedic Surgery, Vita-Salute San Raffaele University, Milan, Italy; 3 Department of Orthopedic Surgery and Traumatology, Faculty of Medicine, Minia University, Minya, 91519 Egypt; 4 IFSTTAR, LBMC UMR_T9406, Claude Bernard University Lyon 1, Université de Lyon, Lyon, France; 5 Department of Orthopedic Surgery and Sport Medicine, FIFA Medical Center of Excellence, Hôpital de la Croix-Rousse, Lyon University Hospital, Lyon, France; 6 Department of Physical Medicine and Rehabilitation, Harvard Medical School, Spaulding Rehabilitation Hospital, Boston, MA 02115 USA; 7 Department of Orthopedic Surgery, Anam Hospital, Korea University, Korea University College of Medicine, Seoul, 02841 Korea, Republic of

**Keywords:** Anterior cruciate ligament, Anterolateral ligament reconstruction, Hamstring autograft, Rotatory knee instability, Pivot-shift, Graft failure, Residual laxity, Meta-analysis

## Abstract

**Background:**

Anterior cruciate ligament reconstruction (ACLR) aims to restore knee stability following anterior cruciate ligament (ACL) rupture. Although hamstring tendon (HT) autografts are the most used, no meta-analysis has specifically evaluated failure rates following primary ACLR with HT in comparisons between standalone ACLR and ACLR combined with anterolateral ligament reconstruction (ALLR).

**Purpose:**

To assess whether adding ALLR to HT autograft ACLR reduces rotatory instability and re-tear rates, and to compare associated complications.

**Methods:**

A systematic electronic search was conducted between January and February 2025 using the Nested Knowledge platform (AutoLit, Nested Knowledge), including data from Cochrane, PubMed, Europe PubMed Central, ClinicalTrials.gov, and the Directory of Open Access Journals, along with supplementary manual searches. Search terms included combinations of “Anterior Cruciate Ligament,” “ACL,” “Anterolateral Ligament,” “Antero-Lateral Ligament,” and “ALL.” Two independent researchers performed screening, and eligible articles underwent full-text review. Inclusion criteria were comparative studies of patients receiving primary single-bundle HT autograft ACLR versus those receiving combined HT ACLR and ALLR. Collected and analyzed data included baseline characteristics; outcomes of pivot shift (PS), laxity measurements, Lachman, and Anterior Drawer tests; complications; re-tear; patient-reported outcome measures (PROMs); and return to athletic activity. The meta-analysis included both prospective and retrospective studies comparing primary HT autograft ACLR with and without ALLR.

**Results:**

A total of 4373 studies were screened, and 12 studies comprising 1274 participants were included. The follow-up duration was 31.87 ± 13.36 months in the ACLR group and 31.01 ± 13.25 months in the ACLR–ALLR group. The mean age was 27.08 ± 3.37 years in the ACLR group and 25.00 ± 3.19 years in the ACLR–ALLR group. The injury-to-surgery interval was 6.86 ± 5.41 months and 6.20 ± 5.49 months in the ACLR and ACLR–ALLR groups, respectively. A postoperative PS odds ratio of 0.20 was observed in the ACLR–ALLR group compared with the ACLR group (*p* < 0.0001), reflecting a relative risk reduction of 81.86% and a number needed to treat of 11.74 to prevent a single event of high-grade pivot shift. Re-tear rates were significantly lower in the ACLR–ALLR group (2.58%) compared with the ACLR group (11.37%) (*p* < 0.00001). Athletic activity, body mass index, sex distribution, complications, and PROMs were comparable between the two groups (*p* > 0.05).

**Conclusions:**

This meta-analysis demonstrates that the addition of ALLR to single-bundle ACLR using HT significantly improves both translational and rotatory stability, reduces the incidence of high-grade pivot shift by 81.86%, and lowers re-tear rates from 11.37% to 2.58%. Notably, these biomechanical benefits are achieved without a corresponding increase in surgical complications, while patient-reported outcomes remain comparable. Although ALLR has traditionally been reserved for selected high-risk patients and elite athletes, the present findings suggest that its potential benefits may extend beyond these populations. Future high-quality randomized trials with standardized outcome reporting are warranted to further refine patient selection and define optimal indications.

## Introduction

The primary goal of anterior cruciate ligament reconstruction (ACLR) is to restore knee stability following anterior cruciate ligament (ACL) rupture and to prevent subsequent injuries while enabling normal daily activities [1–5]. Although the use of ACLR has increased substantially, failure remains relatively common, often presenting as recurrent rotatory instability. To address this issue, adjunct stabilization procedures, such as lateral extra-articular tenodesis (LET) and anterolateral ligament reconstruction (ALLR), have been incorporated into ACLR protocols [6–8].

Graft selection is considered a key factor influencing ACLR durability. Among available options, hamstring tendons (HT)—comprising the gracilis and semitendinosus—represent the most widely adopted graft worldwide [9] owing to their accessibility, mechanical strength, which ranges from 2,160 ± 157 N [10] to over 4000 N [11], and their biological integration [12, 13]. These characteristics have been associated with improved outcomes [14]. However, HT grafts have been associated with increased residual rotational laxity compared with other graft types, which may contribute to persistent pivot shift and has driven interest in adjunctive anterolateral stabilization techniques [6–8, 15].

Failure may ensue owing to a variety of factors, such as graft choice and stabilization technique, complicating the identification of clear predictive factors [16]. Consequently, recent studies have increasingly sought to assess the determinants of ACLR failure, addressing ALLR particularly in revision settings. Nevertheless, limited evidence supports the use of combined ACLR–ALLR as a primary strategy, highlighting the need for further investigation [1, 5–7].

Therefore, this study aims to collect uniform, high-quality data on patients who underwent primary single-bundle ACLR using HT autografts, with or without ALLR, and to evaluate the primary outcome of failure, defined as recurrent rotatory instability, measured by high-grade pivot shift (PS). Secondary outcomes include re-tear rates, high-grade anterior drawer (AD) and Lachman tests, side-to-side differences (STSD), complications (including pain, screw malposition, infection, and arthrofibrosis), and patient-reported outcome measures (PROMs), comprising the International Knee Documentation Committee (IKDC) Subjective Knee Evaluation Form, Lysholm score, and Tegner Activity Scale in standalone ACLR versus ACLR–ALLR groups. The authors hypothesize that combining ALLR with ACLR improves rotatory stability and thereby reduces the risk of ACLR failure and revision compared with standalone ACLR.

## Materials and methods

A systematic electronic search was conducted between January and February 2025 using the Nested Knowledge platform (AutoLit, Nested Knowledge), with data extracted from Cochrane, PubMed, Europe PubMed Central, ClinicalTrials.gov, and the Directory of Open Access Journals, as well as additional free web searches. The search terms included combinations of “Anterior Cruciate Ligament,” “ACL,” “Anterolateral Ligament,” “Antero-Lateral Ligament,” and “ALL.” Abstract screening was performed independently and in parallel by two researchers, and full-text review was conducted for articles deemed eligible for inclusion. Studies were included if they compared patients undergoing primary ACLR using HT autografts with those undergoing ACLR in combination with ALLR. Studies involving LET, nonhamstring ACL grafts, revision ACLR, previous knee surgeries, or those that were noncomparative, biomechanical, anatomical, or cadaveric were excluded.

**Fig. 1:**
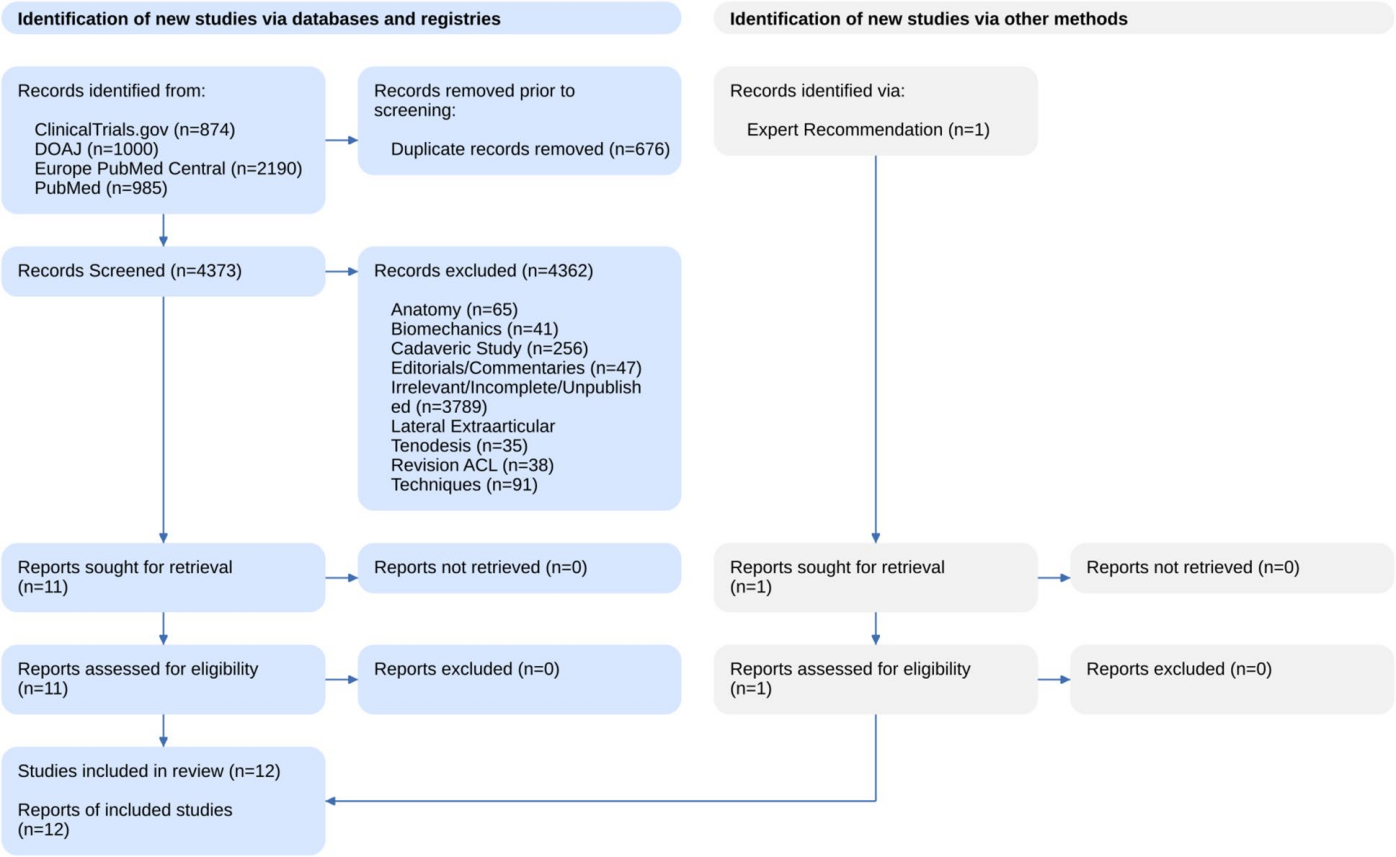
PRISMA 2020 flowchart illustrating the study selection process

A total of 4373 studies were screened, and 12 studies were included [17–28]. The inclusion process is illustrated in the Preferred Reporting Items for Systematic reviews and Meta-Analyses (PRISMA) 2020 flowchart [29] (Fig. 1), and the included studies are presented in Table 1.

**Table 1:** Summary of included studies in the present meta-analysis

Study	Patient number per group/total		
	ACLR (*n*)	ACLR–ALLR (*n*)	Total (*n*)
Zhang 2016	20	20	40
Ibrahim 2017	50	53	103
Sonnery-Cottet 2017	167	221	388
Helito I 2018	68	33	101
Helito II 2019	60	30	90
Hamido 2021	52	50	102
Ortiz de Montellano 2022	52	50	102
Mogos 2023	26	32	58
Lee DW 2023	39	39	78
Torkaman 2024	21	20	41
Gonnachon 2024	42	41	83
Lee JH 2024	26	24	50
Total	623 (48.9%)	651 (51.1%)	1274

Demographic and clinical data—including patient sex, age, body mass index (BMI), follow-up duration, athletic activity level, ACLR technique, PS grade, laxity measurements, Lachman grade, AD grade, complications, re-tear, failure (PS grade), time from injury to surgery, and PROMs such as the IKDC score, Tegner Activity Scale, and Lysholm score—were collected and analyzed.

**Fig. 2:**
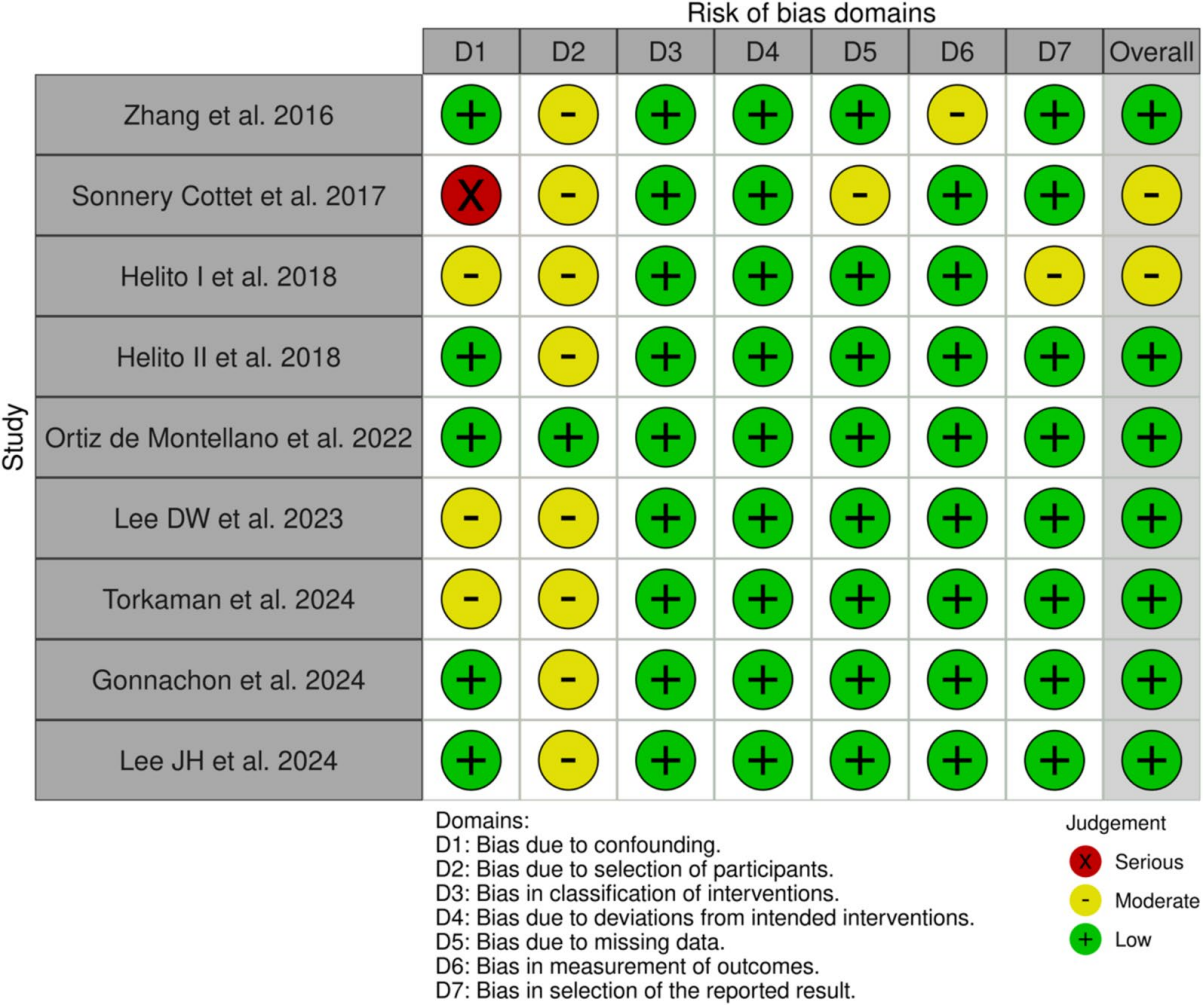
Risk of bias assessment for the included studies using the ROBINS-I tool

**Fig. 3:**
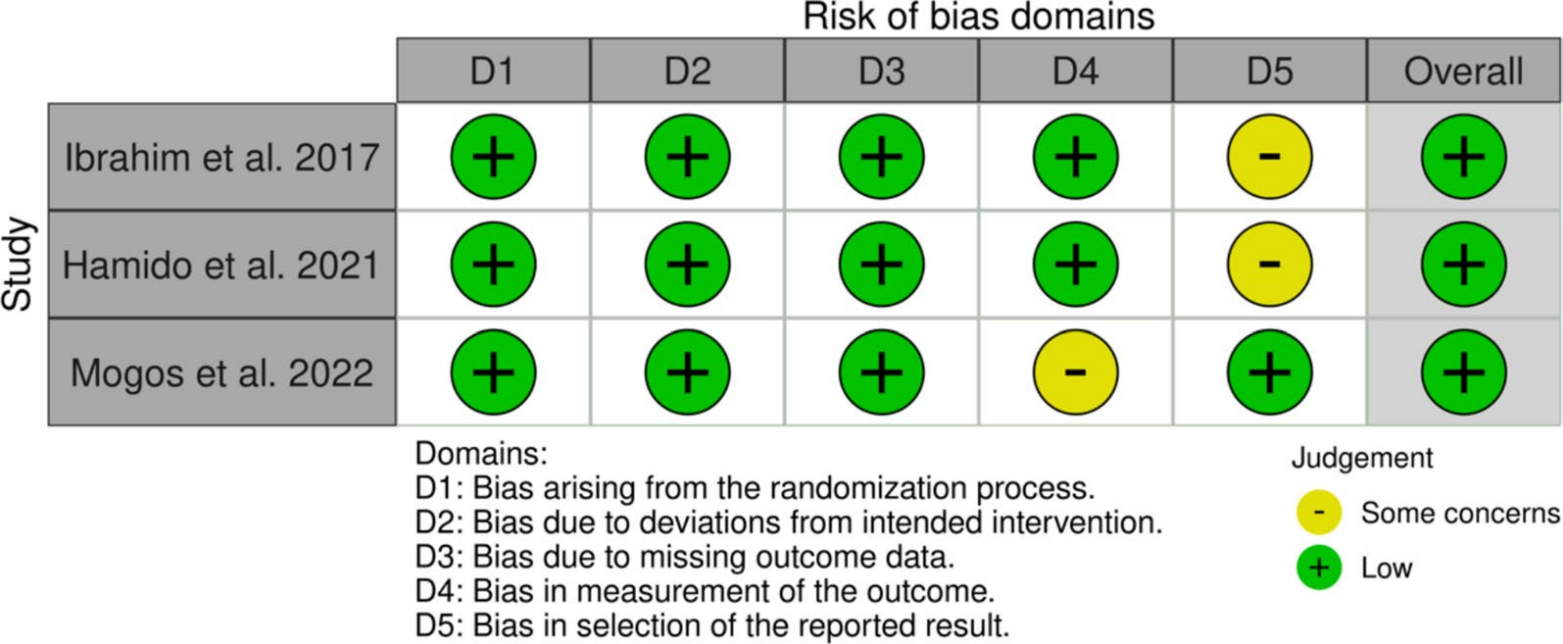
Risk of bias assessment for randomized controlled trial studies using the RoB-2 tool

**Fig. 4:**
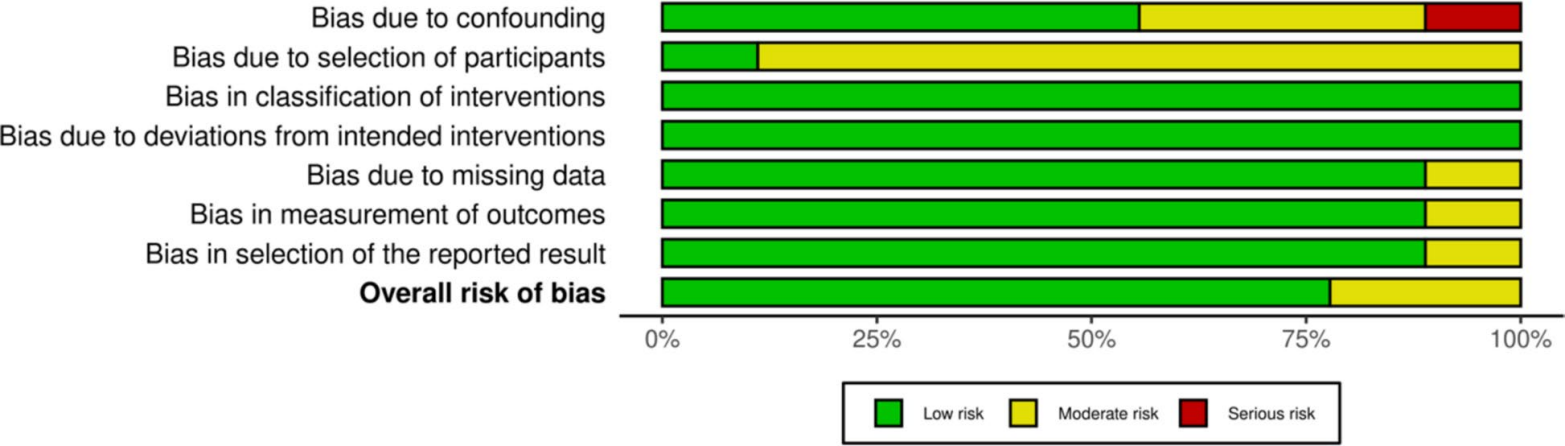
Overall risk of bias summary based on ROBINS-I assessments

**Fig. 5:**
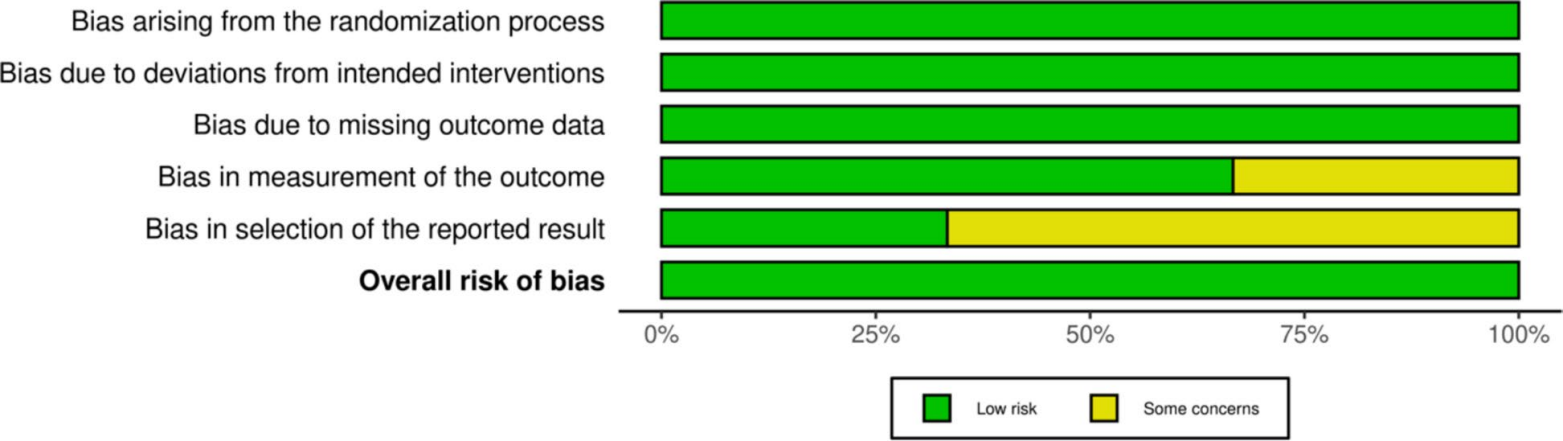
Overall risk of bias summary based on ROB-2 assessments

Risk of bias assessment was independently conducted for all included studies by two investigators (A-M.A. and E.A.E.) using the ROBINS-I tool for retrospective studies and RoB-2 tool for randomized controlled trials. Risk of bias analysis results are illustrated in Figs. 2, 3, 4, 5. In cases of disagreement, third and fourth investigators (S.Y. and K-M.J.) were consulted to reach consensus. Data were organized in Excel spreadsheets, and statistical analyses were performed using RevMan 5.4 [30] and the Social Science Statistics Chi-Square Test Calculator. The Student’s *t*-test was applied to continuous variables, and the Chi-square test was used for categorical variables. Risk ratios, odds ratios, absolute and relative risks, number needed to treat (NNT), and risk differences were calculated and presented in forest plot analyses.

## Results

**Fig. 6:**
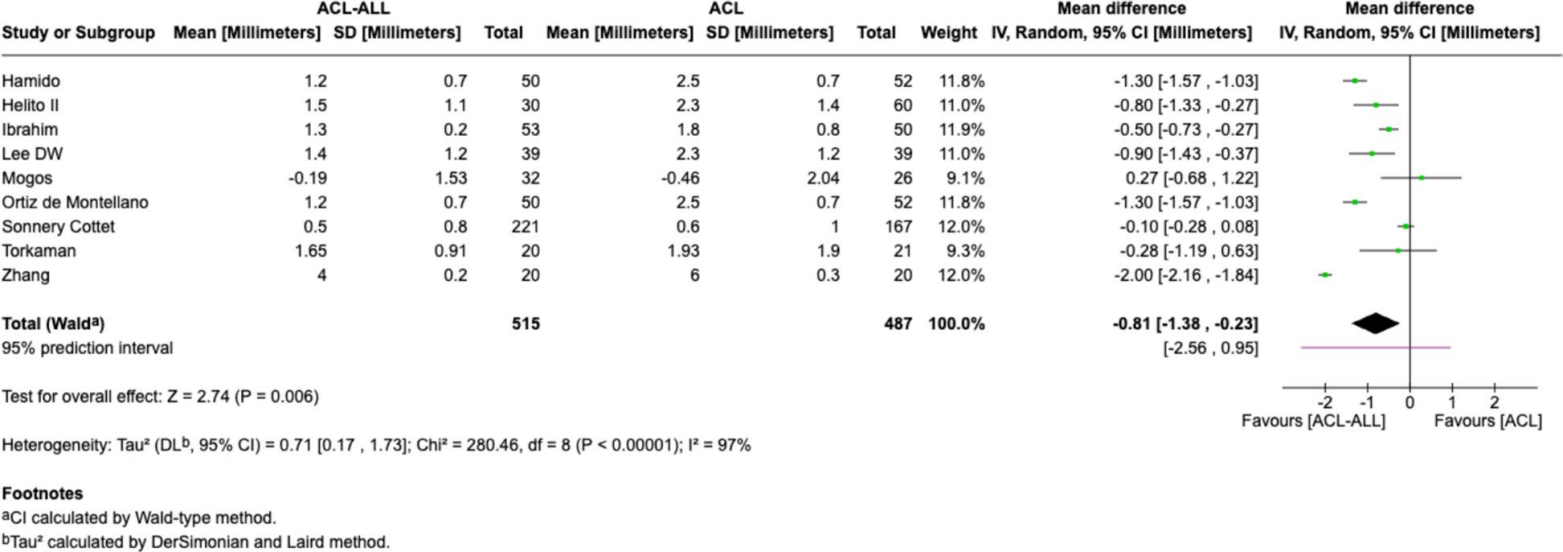
Forest plot illustrating the side-to-side-difference (STSD) parameter

**Fig. 7:**
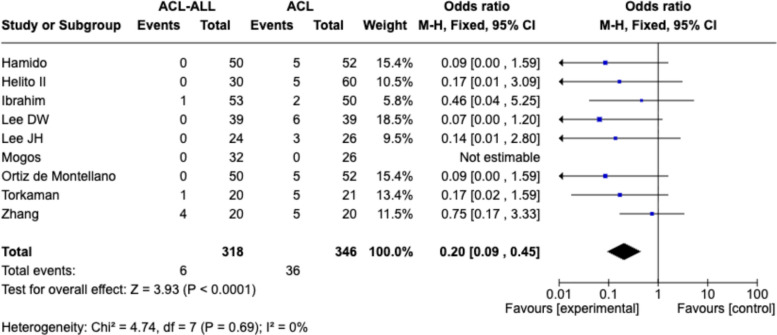
Forest plot illustrating the pivot shift outcome

**Fig. 8:**
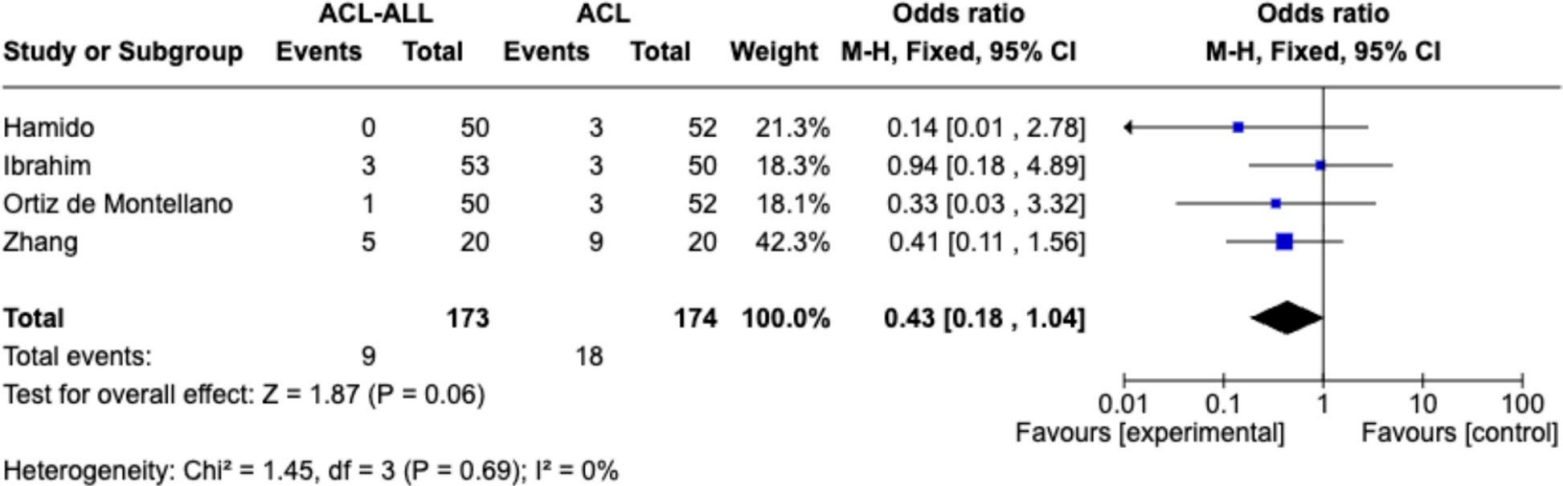
Forest plot illustrating the anterior drawer test outcome

**Fig. 9:**
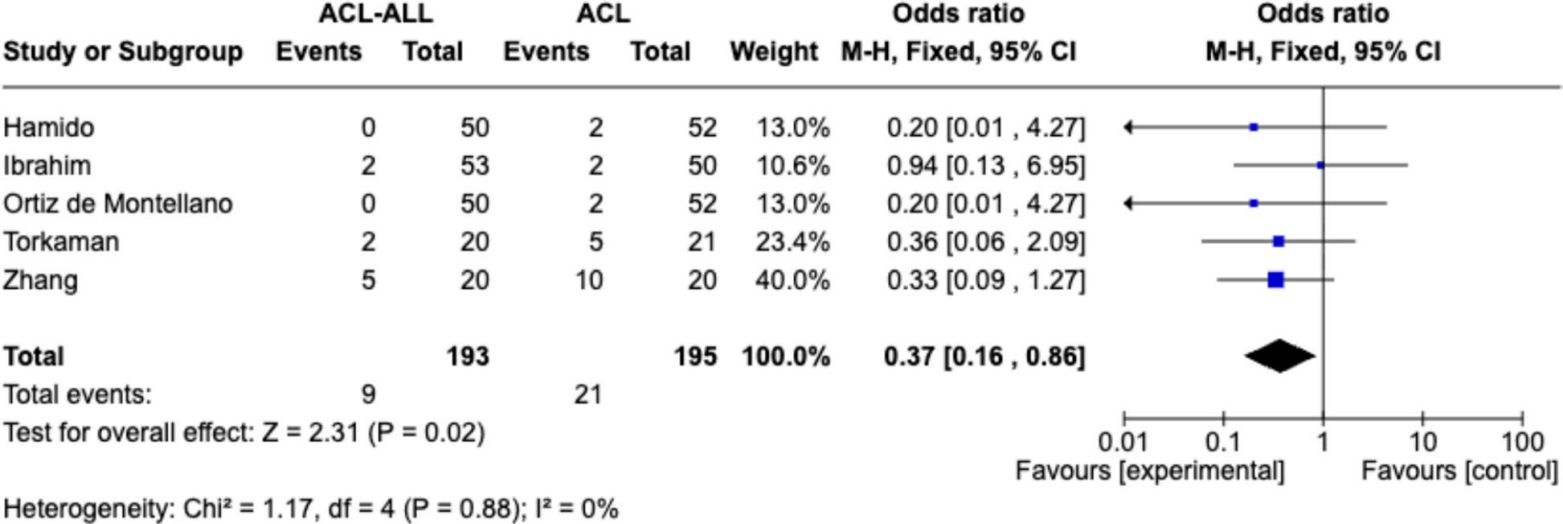
Forest plot illustrating the Lachman test outcome

**Fig. 10:**
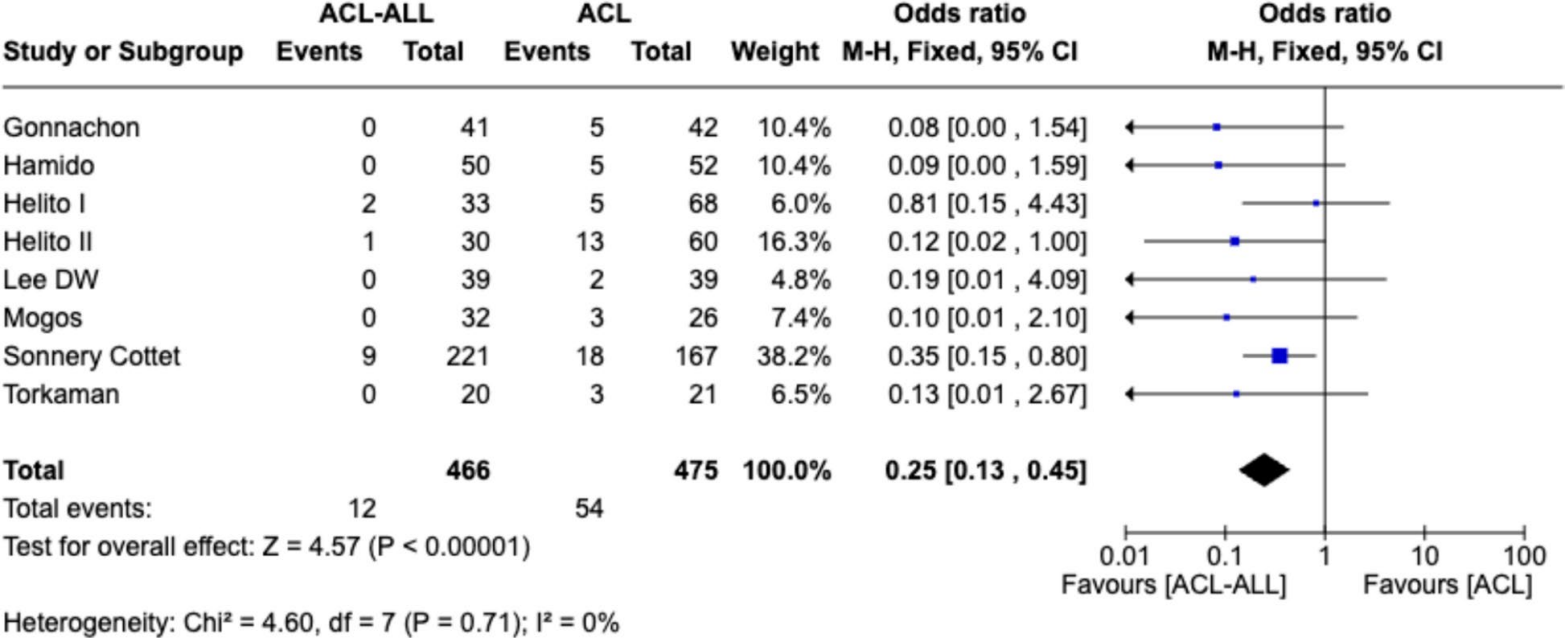
Forest plot illustrating re-tear outcome

**Fig. 11:**
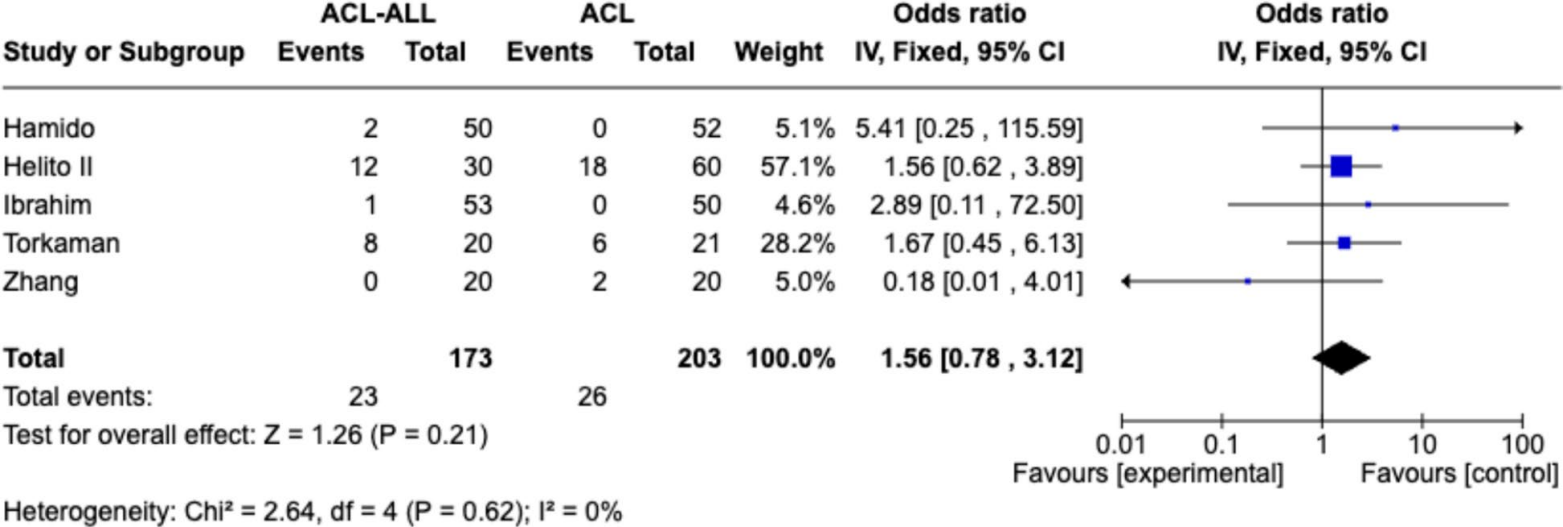
Forest plot illustrating the complication outcome

Results are presented in three subcategories: baseline characteristics (Table 2), outcome data (Table 3), and PROMs (Table 4). Baseline characteristics include age, sex, BMI, follow-up duration (months), and injury-to-surgery interval (months). Outcome data include the incidence of high-grade pivot shift, AD and Lachman tests, STSD, re-tear rates, and complication rates. PROMs comprise the IKDC score, Lysholm score, and Tegner Activity Scale (Figs. 6, 7, 8, 9, 10, 11).

**Table 2:** Patient baseline characteristics

Baseline characteristics			
Parameters	ACLR	ACLR–ALLR	*p*-Value
Age (years)	27.08 ± 3.37	25.00 ± 3.19	< 0.0001*
Male (%)	70.05%	71.33%	0.2268
BMI	23.62 ± 2.66	23.52 ± 2.72	0.7671
FU (months), range	31.87 ± 13.36 (12–60)	31.01 ± 13.25 (12–60)	0.2608
Injury-to-surgery interval (months)	6.86 ± 5.41	6.20 ± 5.49	0.0554
Sex distribution and Athlete prevalence (%)	46.39% (258 M/122F)	53.60% (306 M/133F)	0.5771

BMI: body mass index, FU: follow-up (months)

**Table 3:** Clinical outcome data

Outcome data			
Outcome	ACLR	ACLR–ALLR	*p*-Value
Anterior drawer (events/total)	18/174	9/173	0.06
Lachman (%)	21/195 (10.77%)	9/193 (4.66%)	0.02*
STSD (mm)	1.80 ± 1.36	1.04 ± 0.65	= 0.006, I^2^ = 97%
Pivot Shift (%)	36/346 (10.40%)	6/318 (1.89%)	< 0.0001*
Re-tear (%)	54/475 (11.37%)	12/466 (2.58%)	< 0.00001*
Complications (events/total)	26/203 (12.8%)	23/173 (13.29%)	0.21

Comparison of postoperative outcomes between the ACLR and ACLR–ALLR groups. PS, AD, and Lachman were considered only when graded above II. STSD: side-to-side difference (in millimeters)

**Table 4:** Comparison of functional outcome scores between the ACLR and ACLR–ALLR groups

PROMs data			
Measure	ACLR	ACLR–ALLR	*p*-Value
IKDC	84.60 ± 9.86	85.04 ± 11.05	0.4023
Lysholm	90.40 ± 8.14	90.76 ± 9.11	0.4713
Tegner Activity Scale	6.73 ± 1.42	6.90 ± 1.56	0.0725

PROMs: patient reported outcome measures, IKDC: International Knee Documentation Committee Subjective Knee Evaluation Form

### Baseline characteristics

A total of 1274 patient records were included and analyzed. Sex was reported in 11 of the 12 included studies, encompassing 1153 patients: 338 females (29.31%) and 815 males (70.69%). Age was reported in 1123 cases. The weighted mean age was 27.08 ± 3.37 years in the ACLR group and 25.00 ± 3.19 years in the ACLR–ALLR group. The age difference was statistically significant (*p* < 0.0001). A study level meta-regression was performed using mean age difference between the two groups as a covariate demonstrating no significant association between age difference and failure risk (β = 0.23 per year, *p* = 0.43) while the protective effect of ACLR–ALLR remained significant. No statistically significant differences were found between the two groups in terms of sex distribution, BMI, follow-up duration, injury-to-surgery interval, or athlete prevalence.

### Outcome data

Statistically significant differences were observed between the ACLR and ACLR–ALLR groups in PS, Lachman test, STSD, and graft re-tear rates (*p* < 0.05). By contrast, high-grade AD test (*p* = 0.06) and complication rates (*p* = 0.21) were comparable between groups.

Pivot shift: postoperative high-grade pivot shift (grade II or above) was assessed in nine studies. The pooled odds ratio (OR) was 0.20 (95% confidence interval [CI]: 0.09–0.45) in the ACLR–ALLR group compared with the ACLR group (*p* < 0.0001, *Z* = 3.93, I^2^ = 0%). High-grade pivot shift was observed in 36 of 346 patients (10.40%) in the ACLR group and 6 of 318 patients (1.89%) in the ACLR–ALLR group. This corresponds to a relative risk of 0.18 and a relative risk reduction (RRR) of 81.86%, an absolute risk reduction (ARR) of 8.52%, and a NNT of 11.74 to prevent one case of high-grade pivot shift. A separate meta-regression using mean age as a covariate was performed and did not demonstrate a statistically significant association between age and pivot-shift reduction (β = 0.25 per year, *p* = 0.066). However, the *p*-value approaches statistical significance, suggesting a potential trend that may become evident in larger samples. The protective effect of ACLR–ALLR remained significant. Sensitivity analyses were performed to assess the robustness of the findings, including leave-one-out analyses, and exclusion of the largest and outlying studies, and comparison of fixed and random-effect models. Across all analyses, the direction of effect consistently favored ACLR–ALLR, with no material change in statistical significance. ALL Fixation Angle an exploratory subgroup analysis was performed to evaluate the association between ALL fixation angle and pivot shift outcomes. Studies were categorized into flexion-based (20–30°) [17, 18, 25] and extension-based (near full extension) subgroups [21, 24]. Both subgroups demonstrated a reduction in high-grade pivot shift in favor of combined ACLR–ALLR. The flexion-based subgroup showed a statistically significant effect (OR = 0.31, *p* = 0.035, I^2^ = 0%) whereas the extension-based subgroup demonstrated a similar trend (OR = 0.17), although this did not reach statistical significance. Although no statistically significant difference was observed between the subgroups (*p* = 0.30), this finding should be interpreted with caution, as the extension-based subgroup included only two studies, one of which reported zero events [21]. Anterior Drawer test of high grade was not statistically significant between the two groups unless excluding Ibrahim et al. study in both fixed and random-effect models through sensitivity analysis. Lachman test Grade II or higher Lachman test results were reported in five studies, with 21 events among 195 ACLR patients (10.77%) and 9 events among 193 ACLR–ALLR patients (4.66%). The odds ratio was 0.37 (95% CI: 0.16–0.86, p = 0.02). The ARR was 6.11%, with an NNT of 16. Sensitivity analysis maintained direction and statistical significance except when using random effect whilst excluding Torkaman et al. study. Residual laxity STSD was reported in nine studies, comprising 487 patients in the ACLR group and 515 in the ACLR–ALLR group. The weighted mean STSD between the two groups was 0.81 mm. A Student’s *t*-test revealed a statistically significant difference in residual laxity between groups (*p* < 0.0001), investigation using meta-analytic approach indicated significant difference (*p* = 0.006), however, STSD parameter presented high heterogeneity (I^2^ = 97%). Re-tear was reported in eight studies including 941 patients (475 in ACLR and 466 in ACLR–ALLR). The incidence of re-tear was significantly lower in the ACLR–ALLR group (2.58%) compared with the ACLR group (11.37%) (*p* < 0.00001). Forest plot analysis confirmed a significant reduction in rupture risk, with a relative risk reduction of 77.33%, an odds ratio of 0.25 (95% CI: 0.13–0.45), *p* < 0.00001, I^2^ = 0%), and an NNT of 11.4. Sensitivity analyses were performed to assess the robustness of re-tear reduction in ACLR–ALLR, using leave-one-out analyses, and comparison of fixed and random-effect models. Across all analyses, the direction of effect consistently favored ACLR–ALLR, with no change in statistical significance.

### PROMs

On the basis of weighted averages, the IKDC scores were 84.60 ± 9.86 in the ACLR group and 85.04 ± 11.05 in the ACLR–ALLR group; the difference was not statistically significant (*p* = 0.4023). The Tegner Activity Scale showed averages of 6.73 ± 1.42 in the ACLR group and 6.90 ± 1.56 in the ACLR–ALLR group (*p* = 0.0725). The weighted mean Lysholm scores were 90.40 ± 8.14 and 90.76 ± 9.11 in the ACLR and ACLR–ALLR groups, respectively (*p* = 0.4713).

## Discussion

These findings reinforce the clinical relevance of restoring rotational stability as a key determinant of ACLR success. The main findings of this meta-analysis indicate that combining ALLR with single-bundle HT autograft ACLR significantly improves rotatory stability, reduces graft re-tear and residual laxity (*p* < 0.0001), without a corresponding increase in complication rates. Specifically, this analysis indicated that ALLR addition resulted in a RRR of 81.86% in high-grade pivot shift, with an NNT of 11.74 (*p* < 0.00001). Moreover, the combined approach significantly reduced re-tear rates (2.58% versus 11.37%, *p* < 0.00001) and the incidence of high-grade Lachman test results (10.77% versus 4.66%, odds ratio [OR] = 0.37, *p* = 0.02).

The focus on hamstring tendon (HT) autografts in the present analysis warrants further contextualization, HT grafts were specifically selected due to their widespread clinical use and their known association with increased residual rotational laxity compared with bone–patellar tendon–bone (BPTB) grafts. This characteristic makes HT particularly relevant when evaluating the potential benefit of adjunctive anterolateral stabilization. While the above findings are specific to HT grafts, it is plausible that the synergistic effect of ALL reconstruction may extend to other graft types, such as quadriceps tendon or allografts, particularly in patients with high-grade rotational instability. However, due to the limited and heterogeneous data available for these graft types, this could not be directly assessed and remains an area for future investigation.

### Pivot shift

PS serves as a critical clinical assessment both preoperatively and postoperatively. It not only informs surgical indication but also acts as a key predictor of success during follow-up evaluations [31]. Given its clinical relevance, PS was designated as the primary outcome measure in this analysis. Although many clinicians prioritize graft re-tear as a principal concern, objective measures of rotatory instability such as PS may better reflect functional failure in ACLR [32, 33].

In this meta-analysis, the addition of ALLR to ACLR significantly reduced the incidence of high-grade pivot shift events to 1.89%, compared with 10.40% in standalone ACLR, corresponding to an RRR of 81.86% and an NNT of 11.74 to prevent one high-grade pivot shift event (Fig. 7). These results align with the findings of Jacquet et al. [34], who reported that approximately 24% of patients undergoing ACLR developed high-grade postoperative PS, even when LET was performed. Although our findings are consistent with theirs, differences in graft selection may explain the higher prevalence of high-grade pivot shift reported in their study. Similarly, Mao et al. [35] documented a high-grade pivot shift incidence of 32.6% (163/500) following ACLR. On the basis of these comparisons, we believe that combining ACLR with ALLR offers superior rotatory control compared with alternative techniques, such as LET, where the incidence of high-grade pivot shift ranges between 24% and 28.83% [34, 35].

In the present analysis, although patients in the ACLR–ALLR group were significantly younger, the observed improvement in rotatory stability was not attributable to age. Prior research suggests that each additional year of age is associated with a 9% reduction in the risk of ACLR failure potentially biasing against the combined technique. Contrary to expectations, failure rates remained higher in the older, isolated ACLR group compared with the younger ACLR–ALLR group, suggesting that the addition of ALLR may provide effects across age groups, however, this finding should be interpreted with caution given the lack of statistical significance in meta-regression analysis and effects in older patients that may be actually underestimated [36]. Consequently, a meta-regression using mean age as a covariate was performed showing a nonsignificant statistical association between age and pivot-shift reduction while the protective effect of ACLR–ALLR remained significant independent of age.

Although re-injury is a known possibility, high-grade pivot shift may also occur in the absence of re-tear. This could be partially explained by graft elongation or insufficient ligamentization at the bone–tendon interface, a biological process that typically requires up to 2 years to complete following ACLR without adjunctive procedures [37, 38]. Additionally, despite rotatory stability, the long-term implications for consequent osteoarthritis remain uncertain. Osteoarthritis may develop following ACL injury regardless of surgical treatment; however, the primary goal of reconstruction is to restore joint stability and reduce secondary intra-articular damage, particularly meniscal and chondral lesions as persistent rotational instability has been associated with abnormal joint loading and may contribute to degenerative progression. Therefore, although combined ACL and anterolateral reconstruction cannot be expected to prevent osteoarthritis, improved control of rotatory instability may help preserve joint integrity and potentially delay degenerative changes.

### Re-tear

In this meta-analysis, similar to the pivot shift outcome, the odds of re-tear were approximately four times lower when ACLR was combined with ALLR, corresponding to a relative risk reduction of 77.33%. Re-tear rates were 11.37% in the ACLR group and 2.58% in the ACLR–ALLR group, with extremely strong statistical significance (*p* < 0.00001). These findings reinforce previous results, in which ACLR alone was associated with a 12% re-tear incidence at 5-year follow-up [32], compared with 3.4% in patients treated with combined ACLR–ALLR [39, 40].

The present findings are consistent with those of Sonnery-Cottet et al., who reported a reduced failure rate of 3.5% in the ACLR–ALLR group compared with 17.4% in the standalone ACLR group among high-risk patients [40]. However, their study included patients who received various types of grafts, including different HT configurations and bone–patellar tendon–bone grafts, which may have contributed to the nonuniform failure rates observed in the standalone ACLR cohort.

Re-tear is considered important in all patient groups and particularly critical in athletes, given the substantial reduction in return-to-sport rates following ACLR. This impact becomes even more pronounced when revision procedures are required [41–44].

### STSD

Residual laxity was evaluated using STSD, with measurement methods including KT-1000, KT-2000, and Rolimeter devices. Overall, nine studies reported STSD, comprising 487 patients in the ACLR group and 515 in the ACLR–ALLR group (Fig. 6). Although the difference is statistically significant (*p* < 0.0001 using Students *t*-test and *p* = 0.006 in meta-analytic analysis), the interpretation of this finding is limited by substantial heterogeneity (I^2^ = 97%). This high level of heterogeneity likely reflects variability in measurement techniques, surgical protocols, and reporting standards across studies. Furthermore, the weighted mean difference of 0.81 mm is of limited clinical significance, suggesting that ALLR primarily influences rotatory rather than translational stability [45]. These findings should be interpreted with caution, and greater emphasis should be placed on the more consistent and clinically meaningful improvements observed in rotatory stability.

### Lachman test

Our results indicate that the addition of ALLR to ACLR significantly enhances anterior knee stability, as evidenced by the lower incidence of high-grade Lachman test outcomes (10.77% versus 4.66%), with an odds ratio of 0.37 (95% CI: 0.16–0.86, *p* = 0.02), representing a 63% reduction in anterior laxity. These findings are consistent with previous studies [19], which reported high-grade Lachman results in 5.9% of ACLR–ALLR patients compared with 10–12% in those undergoing standalone ACLR [20].

Interestingly, the NNT to prevent a high-grade Lachman test result (16) is higher than that required to prevent a pivot shift event (11.74), highlighting a clinical dissociation between translational and rotatory laxity. This observation reinforces biomechanical evidence suggesting that ALLR primarily addresses the rotatory component of knee stability, rather than anterior–posterior translation (*p* = 0.06). The authors propose that the observed Lachman–anterior drawer (AD) dissociation may result from a combination of methodological and biomechanical factors, including differing test sensitivities and specificities, variable HT activation during the Lachman test, and the limited number of studies reporting AD outcomes.

### Complications

While the addition of ALLR to ACLR reliably improves rotatory stability, as with all surgical interventions, the clinical decision must weigh potential benefits against procedural risks. The incorporation of ALLR requires an additional surgical incision and introduces risks such as tunnel convergence, among other complications. To objectively address safety concerns, this meta-analysis evaluated complications as discrete adverse events to assess whether the combined procedure increases surgical morbidity. On the basis of these findings, several studies have suggested restricting ALLR to patients with high pivoting demands—such as athletes—or those undergoing revision ACLR [19, 22].

Despite a suggestive trend of the ACLR–ALLR group toward a higher complication rate (OR = 1.56), the incidence of screw malposition, infection, pain, or arthrofibrosis was statistically comparable between ACLR and ACLR–ALLR procedures. These findings further support the rationale for incorporating ALLR into primary ACLR using HT autografts, potentially expanding its indications beyond exclusively high-demand populations.

Pain was the most frequently reported complication, accounting for 84.6% (22/26) of reported events in the isolated ACLR group and 78.3% (18/23) in the ACLR–ALLR group. Persistent concerns regarding potential over-constraint have historically been raised, particularly in LET procedures, and postoperative pain has been suggested to be influenced by surgical technique [16]. In the present analysis, although all ACL reconstruction techniques were reported to be anatomical, heterogeneity was observed in ALLR fixation angles; out of the studies, five reported fixations between 20 and 30° of knee flexion, and three reported ALL fixation near full extension. Notably, Helito II et al. study reported the highest number of pain-related complications and performed fixation near full extension, however, the other studies using the same technique did not address complications in their article. Therefore, although fixation angles may represent a potential factor influencing postoperative pain, available data do not allow definitive conclusions, and this area remains fertile for future investigation.

### PROMs

Despite statistically significant improvements in objective measures of stability and lower graft rupture rates, no corresponding differences were observed in patient-reported outcome measures. Both the IKDC and Lysholm scores fell within the “good” outcome category (*p* > 0.05) [46, 47]. These findings align with prior reports by Torkaman et al. [26], who found no significant differences in IKDC or Tegner scores between the two groups, and by Sørensen et al. [48], who observed no improvement in Tegner scores following ALLR in patients undergoing revision ACLR.

Several factors may explain this apparent discrepancy. First, commonly used PROMs are prone to ceiling effects, particularly in young, athletic, and high-functioning populations, limiting their ability to detect incremental improvements once a “good” functional threshold has been reached. In addition, these instruments are designed to assess general knee function and symptoms and may lack sensitivity to capture high-level, sport-specific activities such as cutting, pivoting, and return-to-sport performance. Consequently, improvements in rotational stability may not be fully reflected in PROM outcomes, despite representing meaningful biomechanical benefits.

Second, the follow-up duration may be insufficient to capture the cumulative clinical impact of subtle instability differences over time. Finally, PROMs reflect the multifactorial nature of patient experience, including psychological, neuromuscular, and activity-related factors, such that improvements in a single biomechanical domain, such as rotational stability, may be diluted within composite scores and remain undetected at the questionnaire level.

### Athletic activity

Athletic activity is a well-known risk factor for ACL injuries and was reported in 819 athletes (64.28% of all patients). Notably, 46.39% of athletes underwent standalone ACLR, while 53.6% received ACLR–ALLR. Among the studies reporting both sex and athletic activity, there was no significant difference in sex distribution between the groups (*p* = 0.5771). However, the interpretation of these results is limited by inconsistent reporting of athletic activity level, type, and intensity across studies.

Although the addition of ALLR introduces additional operative steps, the increase in surgical time is generally modest and, in our experience, typically ranges between approximately 10 and 15 min. This should be considered in the context of its potential to reduce graft failure. From a practical standpoint, preventing even a single revision procedure may offset the additional time and resource utilization associated with ALL reconstruction. While the economic implications remain to be formally established, further studies are warranted to evaluate the cost–benefit profile of combined ACL and ALL reconstruction.

The findings of the current meta-analysis should be interpreted in light of several limitations. The primary focus of this study on HT autograft ACLR limits the generalizability of the findings in other graft types. Further studies are required to determine whether these benefits extend to alternative grafts, including quadriceps tendon and allografts. Despite strict inclusion criteria to avoid graft influence on surgical outcomes [16], heterogeneity in surgical techniques and outcome definitions may have influenced the overall significance and consistency of the results. Moreover, the analysis was conducted in the absence of PROSPERO registration. This research was influenced by partially reported key variables; such as associated lesions, graft diameter, surgical technique, such as fixation angles of ALL, and methods as well as their technique specific complications, and tunnel placement [49, 50], which may directly correlate with specific outcomes such as postoperative stability. An additional important consideration is the inclusion of both randomized controlled trials and retrospective cohort studies within the pooled analysis. Combining different study designs introduces inherent variability in methodological quality and carries a risk of selection bias and residual confounding, particularly from non-randomized data. Owing to the meta-analytic nature of the study, another limitation includes the variability and incomplete reporting of post-operative rehabilitation protocols across studies. Differences in weight-bearing, brace use, and return-to-activity criteria were inconsistently described and could not be quantitatively analyzed. As such, rehabilitation may represent an unmeasured confounder influencing clinical outcomes.

To enhance the robustness and reliability of the analysis, several methodological measures were employed. Although design heterogeneity cannot be completely eliminated, it was partially addressed through separate risk of bias assessments using the RoB-2 tool for randomized studies and the ROBINS-I tool for non-randomized studies. Rigorous screening and inclusion ensured the selection of comparative studies using a single graft type for ACLR (autologous HT), thereby minimizing clinical and surgical heterogeneity. Objective outcomes were prioritized to reduce susceptibility to subjective bias. Statistical heterogeneity was systematically addressed using validated metrics and analytical methods. Additionally, risk of bias was assessed independently by two investigators; in cases of disagreement, a third and fourth reviewer were consulted.

While this study effectively addresses a key gap in the sports medicine literature, it also highlights important avenues for future research. Subsequent investigations should build upon these findings by conducting large-scale trials employing standardized outcome measures and extending the average follow-up duration beyond 30 months to assess mid- to long-term effects. Moreover, consistent reporting of biomechanical factors, graft characteristics, sport type and intensity, and rehabilitation protocols will be essential to identifying predictors of successful outcomes and refining indications for adjunctive anterolateral ligament reconstruction based on patient-specific profiles.

## Conclusions

This meta-analysis demonstrates that the addition of ALLR to single-bundle ACLR using HT significantly improves both translational and rotatory stability, reduces the incidence of high-grade pivot shift by 81.86%, and lowers re-tear rates from 11.37% to 2.58%. Notably, these biomechanical benefits are achieved without a corresponding increase in surgical complications, while patient-reported outcomes remain comparable. Although ALLR has traditionally been reserved for selected high-risk patients and elite athletes, the present findings suggest that its potential benefits may extend beyond these populations. Future high-quality randomized trials with standardized outcome reporting are warranted to further refine patient selection and define optimal indications.

## Data Availability

Data may be provided upon request from the authors.

## Declarations

Ethics approval and consent to participate.
